# Clinical trials registries are under-utilized in the conduct of systematic reviews: a cross-sectional analysis

**DOI:** 10.1186/2046-4053-3-126

**Published:** 2014-10-27

**Authors:** Christopher W Jones, Lukas G Keil, Mark A Weaver, Timothy F Platts-Mills

**Affiliations:** 1 Department of Emergency Medicine, Cooper Medical School of Rowan University, One Cooper Plaza, Suite 152, Camden, NJ 08103, USA; 2 Department of Emergency Medicine, University of North Carolina Chapel Hill, Chapel Hill, USA; 3 Departments of Internal Medicine and Biostatistics, University of North Carolina Chapel Hill, Chapel Hill, USA

**Keywords:** ClinicalTrials.gov, Systematic review, Trials registry, Publication bias

## Abstract

**Background:**

Publication bias is a major threat to the validity of systematic reviews. Searches of clinical trials registries can help to identify unpublished trials, though little is known about how often these resources are utilized. We assessed the usage and results of registry searches reported in systematic reviews published in major general medical journals.

**Methods:**

This cross-sectional analysis includes data from systematic reviews assessing medical interventions which were published in one of six major general medical journals between July 2012 and June 2013. Two authors independently examined each published systematic review and all available supplementary materials to determine whether at least one clinical trials registry was searched.

**Results:**

Of the 117 included systematic reviews, 41 (35%) reported searching a trials registry. Of the 29 reviews which also provided detailed registry search results, 15 (52%) identified at least one completed trial and 18 (62%) identified at least one ongoing trial.

**Conclusions:**

Clinical trials registry searches are not routinely included in systematic reviews published in major medical journals. Routine examination of registry databases may allow a more accurate characterization of publication and outcome reporting biases and improve the validity of estimated effects of medical treatments.

## Background

Systematic reviews are an important means of synthesizing medical research findings to inform medical decision making. The validity of conclusions from systematic reviews depends on the authors’ abilities to identify all previously conducted research relevant to the subject and of sufficient quality to be informative. Because non-publication of original research results that are negative or undesirable threatens the validity of conclusions from systematic reviews, methods have been developed to assess the identified literature for publication bias. These methods, which include the inspection and testing of funnel plots, are now routinely performed and explicitly mentioned in the guidelines for the reporting of systematic reviews [1-3]. However, these methods are imperfect. At best they suggest the presence of unpublished small- or moderately-sized studies based on the assumption that the largest studies on the subject are published, an assumption which may often be incorrect [4]. Empirical analyses indicate that these methods do not reliably detect publication bias [5,6].

Clinical trials registries allow researchers, clinicians, and the general public to learn about clinical trials conducted on a subject regardless of whether the results have been published. Since 2005, the International Committee of Medical Journal Editors (ICMJE) has required that prospective trials involving human participants undergo registration prior to initiating study enrollment as a condition of publication in member journals [7]. Following the passage of the United States Food and Drug Administration Amendments Act (FDAAA) in 2007, prospective trial registration became a requirement under United States law for many interventional clinical trials [8]. Thus, clinical trials registries now form a more comprehensive repository of recently initiated clinical trials. Policies supporting trial registration have been motivated in large part by the belief that consistent pre-registration of clinical trials will help consumers of the medical literature monitor and account for publication bias and other forms of selective reporting [7,9,10].

Awareness of clinical trial results which remain unpublished and unavailable can have important consequences for the conclusions drawn from systematic reviews [11]. Commonly referenced guidelines for the conduct of systematic reviews recommend a search of trials registries as part of a comprehensive search strategy [12,13]. However, whether authors of systematic reviews typically comply with these guidelines is unclear. Our objective was to determine what proportion of authors of systematic reviews recently published in high-impact general medical journals searched clinical trials registries in order to identify relevant unpublished studies.

## Methods

### Data sources

We identified systematic reviews published between July 1, 2012 and June 30, 2013 in six high-impact general medical journals: Annals of Internal Medicine, BMJ, The Journal of the American Medical Association (JAMA), The Lancet, The New England Journal of Medicine, and PLoS Medicine. These journals were chosen because the manuscripts contained therein have a substantial influence on clinical practice and because they also set precedent for medical research methodology. Systematic reviews were identified by searching MEDLINE via PubMed using the following search terms: (Meta-analysis[Publication Type] OR meta-analysis[Title/Abstract] OR meta-analysis[MeSH Terms] OR review[Publication Type] OR search*[Title/Abstract]). This search strategy has been shown to be sensitive for the identification of published systematic reviews [14]. We used the advanced search feature of PubMed to restrict the search to manuscripts with publication dates between July 1, 2012 and June 30, 2013 in the six aforementioned journals.

One study author (CWJ) reviewed the full manuscripts identified during this initial search to determine whether or not each publication included a systematic review. We classified articles as systematic reviews based on previously developed criteria: the manuscript included a clear statement identifying the topic of the review; authors provided a detailed description of the methods and data-sources used to identify evidence included in the review; the manuscript included explicit inclusion and exclusion criteria; and the results included at least one study which met these inclusion criteria [14]. Two study authors (CWJ and LGK) evaluated the included systematic reviews to determine whether the primary goal of each was to assess the effects of an intervention.

### Outcome measures

For each systematic review, two authors (CWJ and LGK) independently reviewed the full text, including appendices and online supplements. The primary outcome was whether the systematic review included a search of at least one clinical trials registry. ClinicalTrials.gov and those trials registries meeting World Health Organization (WHO) Primary Registry Criteria, version 2.1, were included [15] The WHO criteria require that registries pledge to make prospectively registered trial information freely available to the general public. Additional requirements include the ability to track and record any changes made to registry entries, implementation of quality control procedures to ensure the accuracy and completeness of registry data, and management by a not-for-profit agency. Fourteen registries met these requirements at the time of our analysis, including the International Standard Randomised Controlled Trial Number Register and the European Union Clinical Trials Register [15]. Discrepancies between authors with respect to trial registry utilization among the included systematic reviews were resolved by consensus.

### Study variables

When authors indicated that a trials registry search was part of their review protocol, we reviewed the manuscript and all available supplementary materials to determine the number of relevant ongoing or completed studies which were identified as a result of the registry search. For the reviews in which this information was ambiguous or unavailable we emailed the corresponding author to request additional details regarding their search results. We also reviewed full manuscripts and all available supplementary materials to determine whether review authors provided search terms which would allow for the replication of the registry search. We searched each manuscript for a statement from study authors indicating whether the review was compliant with the Preferred Reporting Items for Systematic Reviews and Meta-Analyses (PRISMA) reporting guidelines for systematic reviews. Additionally, we gathered information about study funding and author financial disclosures for each manuscript. Both author support and direct study funding were considered together when categorizing reviews by funding source.

Because clinical trials are more commonly registered than observational studies, registry data are likely to be most relevant to those systematic reviews which evaluate interventions. For this reason, our analysis was limited to those systematic reviews which had a stated primary goal of assessing an intervention in a human population.

### Statistical analysis

Inter-rater agreement for our primary outcome was calculated using Cohen’s kappa. Statistical analyses were performed using SPSS version 20.0 (IBM, Armonk, New York).

## Results

Our PubMed search retrieved 567 records published between July 1, 2012 and June 30, 2013, 177 of which were determined to be systematic reviews (Additional file 1). Of these, 117 (66%) had a stated primary objective of assessing the effects of an intervention and therefore compose our primary study sample. A search of one or more clinical trials registries was described as part of the literature search protocol in 41 (35%) of these 117 manuscripts (Table 1). Subgroup analysis by journal and by funding source showed that there was no subgroup in this sample for which searches of clinical trial registries occurred in more than 50% of the systematic reviews. For 37 reviews, authors reported searching ClinicalTrials.gov either directly or through a registry search portal (International Clinical Trials Registry Platform (ICTRP) or Current Controlled Trials). Nineteen of these 37 reviews included searches of other registries in addition to ClinicalTrials.gov. Four reviews did not specify which trials registries were searched. For the remaining 76 reviews (65%), authors did not report searching a registry.

**Table 1:** Characteristics of systematic reviews published in six major medical journals from July 2012 through June 2013

**Review characteristics**	**All interventional reviews (*n *** **=117)**	**Searched a registry (*n *** **=41)**	**No registry search described (*n *** **=76)**
Journal, *n* (%)			
Annals of Internal Medicine	41	16 (39)	25 (61)
BMJ	37	13 (35)	24 (65)
The Journal of the American Medical Association	11	4 (36)	7 (64)
The Lancet	12	4 (33)	8 (67)
The New England Journal of Medicine	1	0 (0)	1 (100)
PLOS Medicine	15	4 (27)	11 (73)
Funding source, *n* (%)^a^			
Industry	38	17 (45)	21 (55)
NIH^b^/government	81	29 (36)	52 (64)
Other	32	11 (34)	21 (66)
None	13	4 (31)	9 (69)
Manuscript reported PRISMA compliance	38	18 (47)	20 (53)
Number of individual studies included in review, median (range)	30 (5–639)	35 (5–639)	29 (5–379)

^a^Reviews with multiple funding sources are listed within all relevant categories; totals therefore add to more than 100%.

^b^National Institutes of Health.

**Figure 1:**
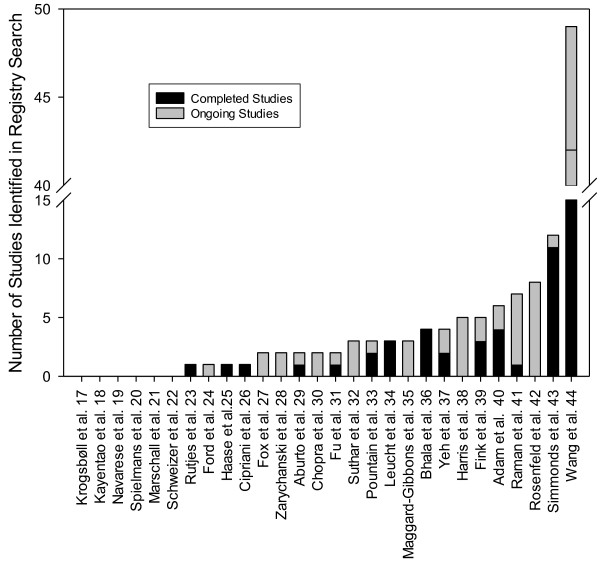
**Registry search results by systematic review **[17]**-**[44]**.***N* =28; one review reported finding “several” relevant ongoing trials.

Detailed registry search results were available for 29 reviews. Authors reported finding at least one relevant completed study as a result of their registry search for 15 of these 29 reviews (52%) and at least one ongoing study for 18 of these reviews (62%; Figure 1). Twenty-three of the 29 reviews (79%) found at least one completed or ongoing study during the registry search. The authors of one of these 23 reviews indicated that they had identified both relevant completed and ongoing studies as a result of their registry search, but they were unable to provide the specific number of studies in each category [16]. Among the reviews for which specific data were available, the median number of relevant studies identified through registry searches was 2 (range 0–49). For six reviews, authors reported finding no relevant ongoing or completed studies as a result of their registry searches. Seven manuscripts provided the specific terms used to perform the registry search.

Cohen’s kappa coefficient for inter-rater reliability with respect to whether systematic reviews included registry searches was 0.98, indicating excellent agreement.

## Discussion

Clinical trials registries were not routinely included as part of the search strategies utilized for this group of systematic reviews published in high-impact general medical journals. Our results show that just 35% of these systematic reviews reportedly incorporated searches from any clinical trials registry. When review authors did search trials registries, however, completed but unpublished studies were identified in approximately half of cases and ongoing studies identified in the majority of cases. These findings suggest that trials registries are an underutilized resource among investigators conducting systematic reviews.

Prior investigations have demonstrated that a substantial proportion of clinical trials are completed but never published [45]. Among trials registered with ClinicalTrials.gov, approximately one in three remain unpublished [46,47], and the rate of non-publication is almost as high for large trials [4]. These estimated rates of non-publication are particularly important given the increasing numbers of trials being registered and conducted. In January of 2005, 12,000 studies had been registered at ClinicalTrials.gov; by October 2013 over 152,000 studies had been registered [48]. Other registries have reported similar growth rates [49]. Thus, clinical trials registries potentially contain a large amount of trial information which is not available in the published literature.

In an effort to make ClinicalTrials.gov more effective at combating publication bias, the United States FDAAA of 2007 expanded the scope of ClinicalTrials.gov via the creation of a results database. The FDAAA mandated that results from nearly all phase II-IV human-subjects trials of FDA-approved drugs or devices conducted within the United States be submitted to ClinicalTrials.gov within one year of the study completion date, regardless of publication status [50]. As utilization of the results database improves, ClinicalTrials.gov will contain outcome data from an increasing number of otherwise unpublished studies. The availability of results will allow a trials registry search to not only inform investigators about the potential for publication bias but also allow for correction of this bias.

Even among registered studies without results available on ClinicalTrials.gov, registry entries are likely to contain information of significant value to the authors of systematic reviews. First, comparing registered studies to published studies allows identification of unpublished trials, and authors of systematic reviews can use the number and sample sizes of unpublished studies in combination with other methods such as funnel plots to better describe the possible impact of publication bias on effect estimates. For example, particular caution should be exercised when drawing conclusions from pooled data when registry searches reveal the existence of unpublished trials with large sample sizes relative to the subset of published trials. Second, even when trial results are published, examining registry entries can help review authors identify outcome reporting bias by allowing them to compare *a priori* planned outcomes to reported outcomes [51,52]. Additionally, registry entries frequently include contact information for study investigators, thereby providing review authors with the opportunity to contact investigators to request information about unpublished or incompletely reported studies. A recent comparison between published trial results and results posted on ClinicalTrials.gov also showed that ClinicalTrials.gov was far more likely than a published manuscript to include a complete accounting of adverse study events [53]. Finally, registry searches may reveal the existence of ongoing or recently completed trials, allowing the authors of systematic reviews to more accurately discuss their results within the context of ongoing research.

Our results are similar to findings from an analysis of Cochrane systematic reviews published between 2008 and 2010, in which trials registry searches were only included in 38% of reviews [54]. This study found that for the reviews in which registry search results were reported, relevant completed or ongoing trials were identified just 45% of the time. In our study, at least one relevant completed or ongoing trial was identified by 79% of the reviews reporting registry search results. One possible explanation for this difference is that the utility of registry searches may be increasing with time as trial pre-registration becomes more commonplace.

Since 2009 the Centre for Reviews and Dissemination has identified ClinicalTrials.gov and other public trials registries as important resources for identifying trial results [55]. Other commonly referenced guidelines for the conduct of systematic reviews, including those produced by the Institute of Medicine and the Agency for Healthcare Research & Quality, have strongly recommended the use of a clinical trials registry search as part of a comprehensive search strategy since 2011 [12,13]. Further, the Cochrane Handbook recommends that Cochrane Review authors search registries in order to assess both for completed but unpublished and for ongoing trials [56]. Despite these recommendations, our results show that trials registries are not routinely utilized by systematic review authors. The PRISMA Statement, which has been endorsed by the World Association of Medical Editors, the Cochrane Collaboration, and numerous other organizations and journals, and provides a guide to the reporting of systematic reviews, does not explicitly advise authors to search trial registries [1]. While PRISMA is intended to be a guide to systematic review reporting, it is probably also used by investigators as a guide to systematic review conduct. As compliance with trial registration requirements and use of the ClinicalTrials.gov database for reporting results increase, revisions to PRISMA that place greater emphasis on incorporating registry data into systematic reviews may be useful in raising awareness among authors of systematic reviews of the potential importance of this resource.

Several limitations should be considered when interpreting these results. Not all systematic reviews address topics which can be studied with a clinical trial, and it is possible that even among the group of reviews which assessed interventions, some review authors decided not to search a trial registry because they thought that there were no relevant trials on the subject. However, registry data may be valuable even for authors conducting systematic reviews of subjects not amenable to clinical trials. Currently over 27,000 observational studies are registered with ClinicalTrials.gov, accounting for 18% of the database’s registry entries, and the authors of observational studies are increasingly being encouraged to prospectively register their studies [48,57]. ClinicalTrials.gov functions as a significant source of registry data from non-interventional studies, and even systematic reviews that examine questions not easily tested with a clinical trial may benefit from registry searches. Additionally, we did not assess the impact of searching industry-sponsored results databases in this analysis, though these databases can also be important sources of unpublished trial data [58].

The external validity of our results may also be limited. We studied systematic reviews published in a group of high-impact general medicine journals; it is possible that different patterns would be observed among other journals. Also, it is probable that some systematic reviews meeting our inclusion criteria were missed by our search strategy. However, the strategy we employed has previously been validated as a sensitive method of identifying systematic reviews, and it is unlikely that any systematic reviews we overlooked would have a substantively different rate of searching trials registries.

## Conclusions

In this sample of recently published systematic reviews in major medical journals, searches of clinical trials registries were not routinely utilized. In the cases where registry searches were performed, authors identified relevant completed or ongoing trials more than three quarters of the time. More consistent use of trials registry databases may improve the identification of publication and outcome reporting biases and increase the validity of estimated effects of medical treatments in systematic reviews.

## Abbreviations

ICMJE: International Committee of Medical Journal Editors; FDAAA: Food and Drug Administration Amendments Act; JAMA: The Journal of the American Medical Association; WHO: World Health Organization; PRISMA: Preferred Reporting Items for Systematic Reviews and Meta-Analyses; ICTRP: International Clinical Trials Registry Platform.

## Competing interests

The authors declare that they have no competing interests.

## Authors’ contributions

All authors contributed to the study concept and design. Data acquisition was performed by CWJ and LGK. Data analysis and interpretation was performed by CWJ, LGK, MAW, and TPM. MAW provided statistical expertise. The initial manuscript was drafted by CWJ, LGK, and TPM; all authors contributed to subsequent revisions. CWJ had full access to all the data in the study and had final responsibility for the decision to submit for publication. All authors read and approved the final manuscript.

## Authors’ information

CWJ is an investigator on a study sponsored by Roche Diagnostics which provides financial support to his department. MAW is supported by the National Center for Research Resources and the National Center for Advancing Translational Sciences through grant UL1 TR001111-01, and TPM is supported by the National Institute on Aging through grant K23 AG038548. These sponsors had no role in the study design; collection, analysis, and interpretation of the data; writing of the report; or in the decision to submit the manuscript for publication.

## Supplementary Material

Additional file 1 **Systematic reviews.** Records published between July 1, 2012 and June 30, 2013 and identified via PubMed search. Click here for file
